# Facile and Versatile Method for Micropatterning Poly(acrylamide) Hydrogels Using Photocleavable Comonomers

**DOI:** 10.1021/acsami.1c17901

**Published:** 2022-01-10

**Authors:** Dimitris Missirlis, Miguel Baños, Felix Lussier, Joachim P. Spatz

**Affiliations:** † Department of Cellular Biophysics, Max-Planck-Institute for Medical Research, Jahnstr. 29, Heidelberg 69120, Germany; ‡ Department of Biophysical Chemistry, Physical Chemistry Institute, Heidelberg University, INF-253, Heidelberg 69120, Germany

**Keywords:** mechanotransduction, integrin ligands, cell−material interactions, photopatterning, traction force microscopy

## Abstract

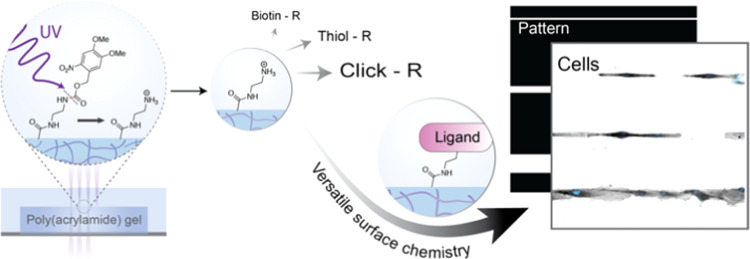

We here present a micropatterning strategy to introduce small molecules and ligands on patterns of arbitrary shapes on the surface of poly(acrylamide)-based hydrogels. The main advantages of the presented approach are the ease of use, the lack of need to prefabricate photomasks, the use of mild UV light and biocompatible bioconjugation chemistries, and the capacity to pattern low-molecular-weight ligands, such as peptides, peptidomimetics, or DNA fragments. To achieve the above, a monomer containing a caged amine (NVOC group) was co-polymerized in the hydrogel network; upon UV light illumination using a commercially available setup, primary amines were locally deprotected and served as reactive groups for further functionalization. Cell patterning on various cell adhesive ligands was demonstrated, with cells responding to a combination of pattern shape and substrate elasticity. The approach is compatible with standard traction force microscopy (TFM) experimentation and can further be extended to reference-free TFM.

## Introduction

Understanding how cells sense and respond to the physical and mechanical properties of their insoluble microenvironment, i.e., their extracellular matrix (ECM), is a major challenge of mechanobiology research. Among different approaches, the ex vivo interrogation of cells on artificial substrates with controlled biophysical and biochemical properties has proven to be a powerful tool to test hypotheses and gain mechanistic insight into mechanosensing and mechanotransduction of living cells. Upon adhesion on a compliant substrate, cells exert traction forces at the sites of attachment, where multiprotein complexes, termed focal adhesions (FAs), assemble.^1,2^ Cell-surface receptors of the integrin family at FAs transmit intracellularly generated forces produced by actomyosin contractility to substrate-immobilized extracellular ligands.^3^ The amplitude and dynamics of these forces depend on substrate viscoelasticity, and in turn determine the tension and force loading rate experienced by mechanosensing proteins present at FAs.^1^ At the same time, cell shape and size additionally control the magnitude and orientation of traction forces, through control of adhesion geometry and actin cytoskeleton organization.^4,5^ Efforts to control cell morphology have revealed how cell confinement affects cell growth,^6^ gene transcription,^7^ and differentiation.^8^ Overall, both cell shape and substrate mechanics control cell physiology, and hence the need to control independently these parameters when designing cell culture substrates. An attractive approach to achieve this goal is to pattern adhesive ligands on viscoelastic substrates so that cells conform to the designed patterns.^9,10^

Hydrogels based on synthetic or natural non-ECM polymers offer the advantage of decoupling ligand presentation from mechanical properties, due to their tunable stiffness and biologically inert background, on which adhesive ligands can be incorporated.^11−15^ Various systems and cross-linking chemistries have been developed to this end, including but not limited to poly(ethylene glycol), alginate, hyaluronan, poly(*N*-isopropylacrylamide), and poly(hydroxyethyl methacrylate) hydrogels.^13−15^ To spatially pattern adhesive ligands on the hydrogel surface,^16,17^ light has proven an excellent means due to a range of developed photocleavable groups and the precision photopatterning allows.^18,19^ Current methodologies for hydrogels photopatterning rely primarily on soft lithography and can be classified into two main categories. The first, most common strategy is based on localized activation of reactive groups or exposure of protein-adsorbing surfaces on the gel surface and subsequent, selective attachment of ligands.^20−28^ Within this category, additionally falls the spatially controlled uncaging of adhesive ligands, which does not require a second reaction step, but prior synthesis and incorporation of caged ligands.^29,30^ The second strategy is based on hydrogel formation on a substrate prepatterned with the ECM protein of choice.^4,31,32^

Among the developed hydrogel systems, polyacrylamide (pAAm) gels remain the most popular choice for cell mechanobiology studies due to their ease of fabrication and established use in traction force microscopy (TFM) studies following decoration with fiducial markers.^22,33−35^ Despite inherent limitations, such as lack of physiological structure and viscoelastic character, pAAm hydrogels offer a reproducible, robust, and stable system that can be readily functionalized with high-molecular-weight adhesive ligands.^35,36^ To pattern such ligands, a variety of micropatterning techniques were developed, or adapted for pAAm,^20−22,31,32^ published protocols for the spatial patterning of this material still suffer from a few drawbacks: (i) a new pattern requires the design and fabrication of a new mask, an expensive and time-consuming process; (ii) current approaches do not allow efficient patterning of small (1–3 kDa) peptide ligands, (iii) aligning multiple, sequential patterns is difficult; and (iv) flexibility and control over ligand presentation remains limited. Consequently, there is room for improved techniques that are more versatile, easy-to-perform, and accessible.

Here, we introduce a facile and versatile method to pattern pAAm hydrogels using UV light, through the co-polymerization of monomers containing light-sensitive caged amine groups. The generated primary amines can be subsequently used to immobilize cell ligands throughout various conjugation methodologies. The presented technique takes advantage of a commercially available micropatterning system on an optical microscope but should be applicable on most standard laser-based microscopy systems of a typical research lab.

## Results and Discussion

### Incorporation and Patterning of Primary Amine Groups in pAAm Hydrogels

**Figure 1:**
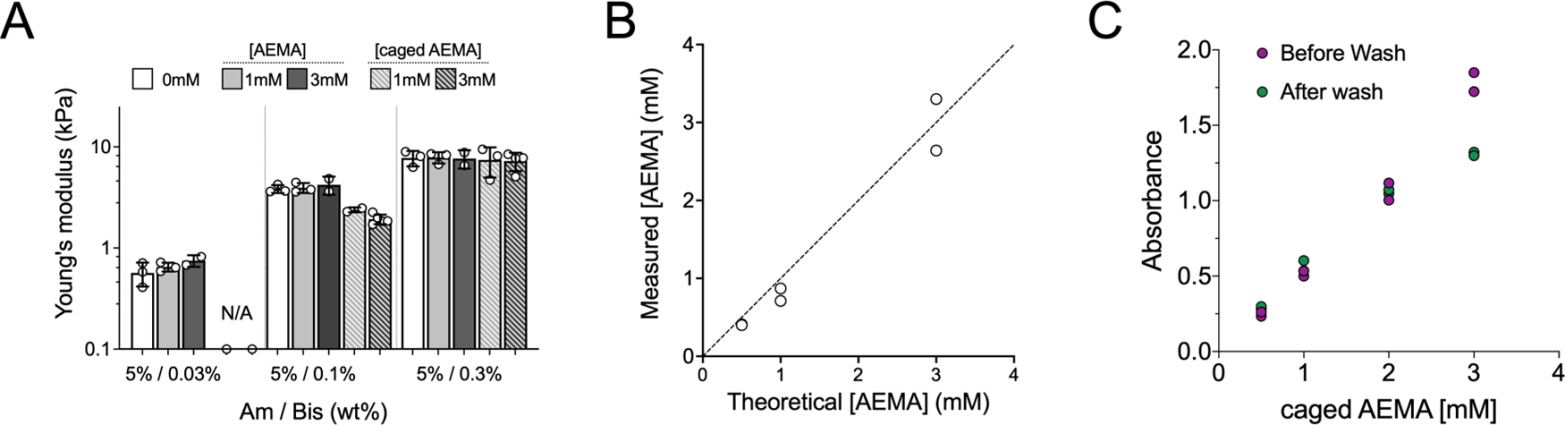
Incorporation of reactive amines in poly(acrylamide) hydrogels. (A) Young’s moduli calculated from atomic force microscopy (AFM) indentation measurements of pAAm hydrogels with indicated nominal concentrations of comonomers. Each data point (open circles) corresponds to a different hydrogel. The bar is the mean from *n* = 2–5 independent batches, and the error bars represent the standard deviation. (B) Incorporation of AEMA in pAAm hydrogels (5% Am/0.1% Bis) calculated using a fluorescamine assay showed quantitative incorporation of AEMA. Each data point corresponds to an independent experiment (*n* = 3 gels/experiment). (C) Incorporation of caged AEMA in pAAm hydrogels (5% Am/0.1% Bis) calculated using UV measurements of precursor solutions and hydrogels following formation and extensive washing. Each data point corresponds to an independent experiment (*n* = 3 gels/experiment).

Poly(acrylamide) (pAAm) hydrogels were prepared via radical cross-linking polymerization of acrylamide (Am) and bis-acrylamide (Bis) (Figure S1). The Am and Bis concentrations determine the cross-linking extent, and hence the stiffness of the resulting pAAm hydrogels.^35^ Hydrogels with Young’s moduli between 0.5 and 10 kPa were obtained for the different formulations prepared, a range that corresponds to most soft tissues found in the body (Figure 1A). To include reactive groups in the polymer network, the introduction of appropriate comonomers in the polymerization mixture has been proposed.^4,20,37,38^ Initial attempts to employ 2-aminoethyl methacrylate as previously reported^39^ were not successful due to monomer insolubility—or fast degradation—of the commercially available chemical in buffered solutions or dimethylformamide (DMF). On the other hand, the incorporation of 2-aminoethylmethacrylamide (AEMA) at concentrations between 0.5 and 3 mM was effective. The presence of nucleophilic primary amines in AEMA-containing hydrogels was quantified using fluorescamine, and the amount of incorporated comonomer increased linearly with precursor concentration, providing control over the number of incorporated reactive groups (Figure 1B). Of note, AEMA incorporation did not affect hydrogel stiffness (Figure 1A).

**Figure 2:**
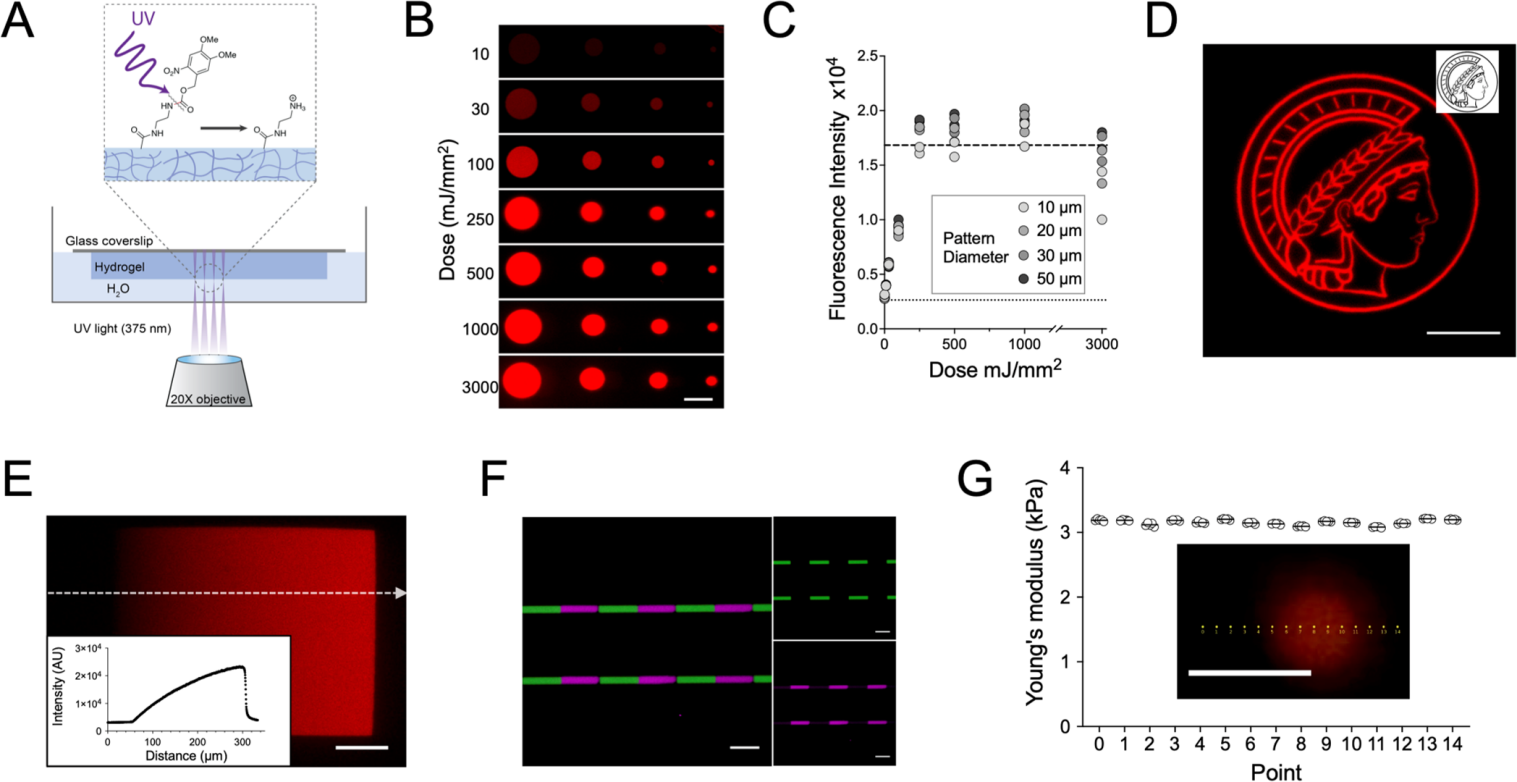
Hydrogel patterning with fluorescent dyes. (A) Schematic representation of the strategy used to pattern poly(acrylamide) hydrogels containing caged AEMA comonomers. A changeable virtual mask is used by the PRIMO patterning system to illuminate selected regions of the hydrogel with UV light and locally deprotect amine groups. (B) Confocal microscopy images of *N*-hydroxysuccinimide (NHS)-AF568 dye coupled to different-sized circular patterns on hydrogels using different light doses. The intensities of the images are normalized. (C) Quantification of mean fluorescence intensity inside patterned circles of different sizes as a function of UV light dose. The dashed line corresponds to the fluorescence intensity of a hydrogel containing the same concentration of AEMA (1 mM) as the caged AEMA in the patterned sample and reacted with the same amount of dye. The dotted line corresponds to a hydrogel without any AEMA and reacted with the same amount of dye. Each data point corresponds to an independent experiment (*n* = 2). (D) Confocal microscopy image of hydrogel patterned with a custom design (inset: binary image) and reacted with NHS-AF568 dye. (E) Confocal microscopy image of hydrogel patterned with a virtual mask exhibiting a linear gradient in gray values and reacted with NHS-AF568 dye; the inset shows the fluorescence intensity profile along the white dahsed line. (F) Confocal microscopy image of a hydrogel initially patterned and reacted with an NHS-AF568 dye (green), washed, patterned again, and reacted with an NHS-Atto647N dye (magenta). (G) Young’s moduli values obtained at different locations on the surface of a hydrogel (5% Am/0.1% Bis; 1 mM caged AEMA), patterned with 250 mJ/mm^2^ UV light and reacted with NHS-AF568 dye. The inset shows the locations of each point overlaid on an epifluorescence image of the hydrogel surface. The values obtained for points 6–10, which are within the pattern, show no difference compared to the ones outside the pattern. Scale bar: 50 μm.

To introduce amines in a spatially controlled manner, a light-sensitive comonomer was synthesized and incorporated in the hydrogels. The commonly used nitrophenyl-containing group (NVOC)^16,40^ was linked to AEMA, thus protecting the primary amine (caged AEMA; Figure 2A). Due to low aqueous solubility, caged AEMA was dissolved in DMF instead of water. Moreover, preliminary studies showed that a higher amount of radical initiation was required for polymerization due to the radical scavenging properties of the nitro group.^41^ The nitrophenyl group absorbs light in the UV range; quantification of comonomer incorporation through absorbance measurements following extensive washing of the gel revealed a monotonic increase in caged AEMA incorporation (Figure 1C). The caged comonomer was quantitatively incorporated, except for the highest concentration tested (3 mM), where approximately 70% of the initial caged AEMA was present in the final hydrogel (Figure 1C). The mechanical properties of the resulting hydrogels were influenced by the presence of the caged comonomer, depending on the cross-linking ratio: for the stiffer hydrogels (0.3% Bis), the obtained Young’s moduli were similar to controls, whereas for hydrogels with lower cross-linking, a reduction in stiffness was observed. For the lower cross-linking concentration (0.03% Bis), the hydrogels obtained were too soft to handle and measure with the AFM setup used.

### Light-Induced Uncaging Enables Spatial Patterning

The caged comonomer was designed such that the cleavage of the carbamate group upon UV irradiation would expose primary amines for further functionalization as schematically shown in Figure 2A. To determine the light exposure required to deprotect the amines of gel-incorporated caged AEMA, circular patterns of 10–50 μm in diameter on the hydrogel surface were exposed to UV light (375 nm) for varying times using a PRIMO micropatterning system. Deprotected amines were then reacted with an NHS-coupled fluorescent dye (AlexaFluor 568). A linear increase in fluorescence intensity within patterned areas was observed up to a laser dose of 250 mJ/mm^2^, followed by a plateau (Figure 2B,C). The plateau value was the same as the one for hydrogels containing AEMA at the same concentration, indicating complete uncaging of caged AEMA at illuminated regions. Nonpatterned areas exhibited similar fluorescence to the negative controls (no AEMA incorporated), demonstrating a lack of uncaging from ambient light in nonilluminated regions. Based on these results, a dose of 500 mJ/mm^2^ was selected for further experiments.

The PRIMO micropatterning system with its associated software also enabled the facile preparation of virtual masks, and thus the patterning of custom designs (Figure 2D), as well as the formation of gradients of immobilized fluorescent labels, through the use of virtual masks with a gradient in gray value (Figure 2E). Patterning of two different dyes in complex, predesigned patterns was also achieved with sequential light exposure, fluorescent labeling, and alignment using the fluorescence of the first pattern (Figure 2F).

Exposure to UV light and deprotection of caged AEMA did not affect the stiffness of the hydrogel surface layer, as determined by AFM indentation measurements. The Young’s modulus was the same within and around patterns, as these were identified by fluorescent labeling with NHS-AF568 (Figure 2G). Of note, reaction with NHS-AF568 labeled the deprotected amines throughout the hydrogel bulk following the UV light path used during patterning (Figure S2). In principle, localized patterning with the hydrogels presented here is also feasible in the *z*-direction using two-photon microscopy.^42,43^

### Hydrogel Functionalization

**Figure 3:**
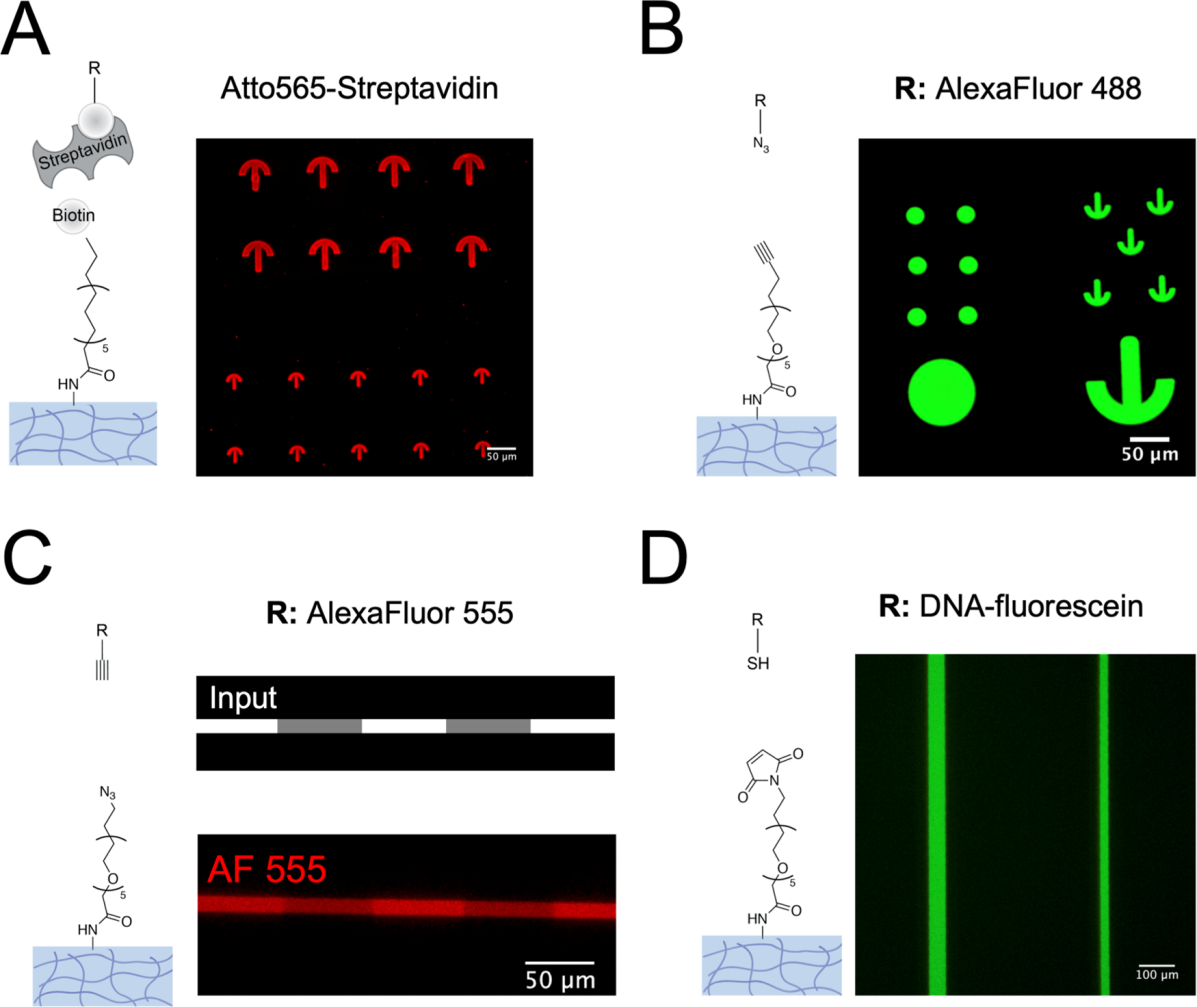
Pattern functionalization on hydrogels via different chemistries. Confocal microscopy images of pAAm hydrogels (5% Am/0.1% Bis) patterned with UV light to uncage locally primary amines and reacted with: (A) first an NHS-biotin linker, followed by incubation with Atto565-labeled streptavidin; (B) first an NHS-PEG-Alkyne linker, followed by a click reaction with an azide coupled to AlexaFluor488; (C) first an NHS-PEG-Azide linker, followed by a click reaction with an alkyne coupled to AlexaFluor 555; and (D) first an NHS-PEG-Maleimide linker, followed by a second reaction with a thiol-containing, fluorescein-labeled DNA fragment. Scale bar (A–C): 50 μm, D: 100 μm.

Having validated the presence of reactive amines on patterned regions (Figure 2), three different chemistries were examined with the aim of introducing spatially patterned functional molecules on the surface of poly(acrylamide) hydrogels (Figure 3). First, deprotected amines in hydrogels were reacted with a heterobifunctional NHS-biotin linker to exploit the robust biotin–streptavidin interaction to immobilize ligands on surfaces. Subsequent incubation with fluorescently labeled streptavidin (100 μg/mL) verified the applicability of this approach to surface pattern hydrogels (Figure 3A). In this case, Atto565-Streptavidin was only present on the hydrogel surface, due to the size-dependent exclusion of the protein from diffusion inside the hydrogel (Figure S2). Second, click chemistry was performed following reaction of patterned hydrogels with a heterobifunctional NHS-PEG_5_-Alkyne linker: an azide-functionalized fluorophore (AlexaFluor488) was used to validate this approach (Figure 3B). Inversely, the azide was immobilized on the patterned hydrogel using a heterobifunctional NHS-PEG_4_-azide linker and patterned regions were labeled with alkyne-functionalized AlexaFluor 555 (Figure 3C). Third, a bifunctional NHS-PEG_2_-maleimide linker was first introduced to react the NHS esters with the amines, followed by coupling to maleimides of a short thiol-containing DNA fragment, labeled with fluorescein (Figure 3D). Due to maleimide degradation in aqueous solutions, these two steps should be performed in quick succession. The above results highlight the versatility of patterning this type of hydrogels with commonly used bioconjugation chemical strategies.

### Cell Patterning on Hydrogels

**Figure 4:**
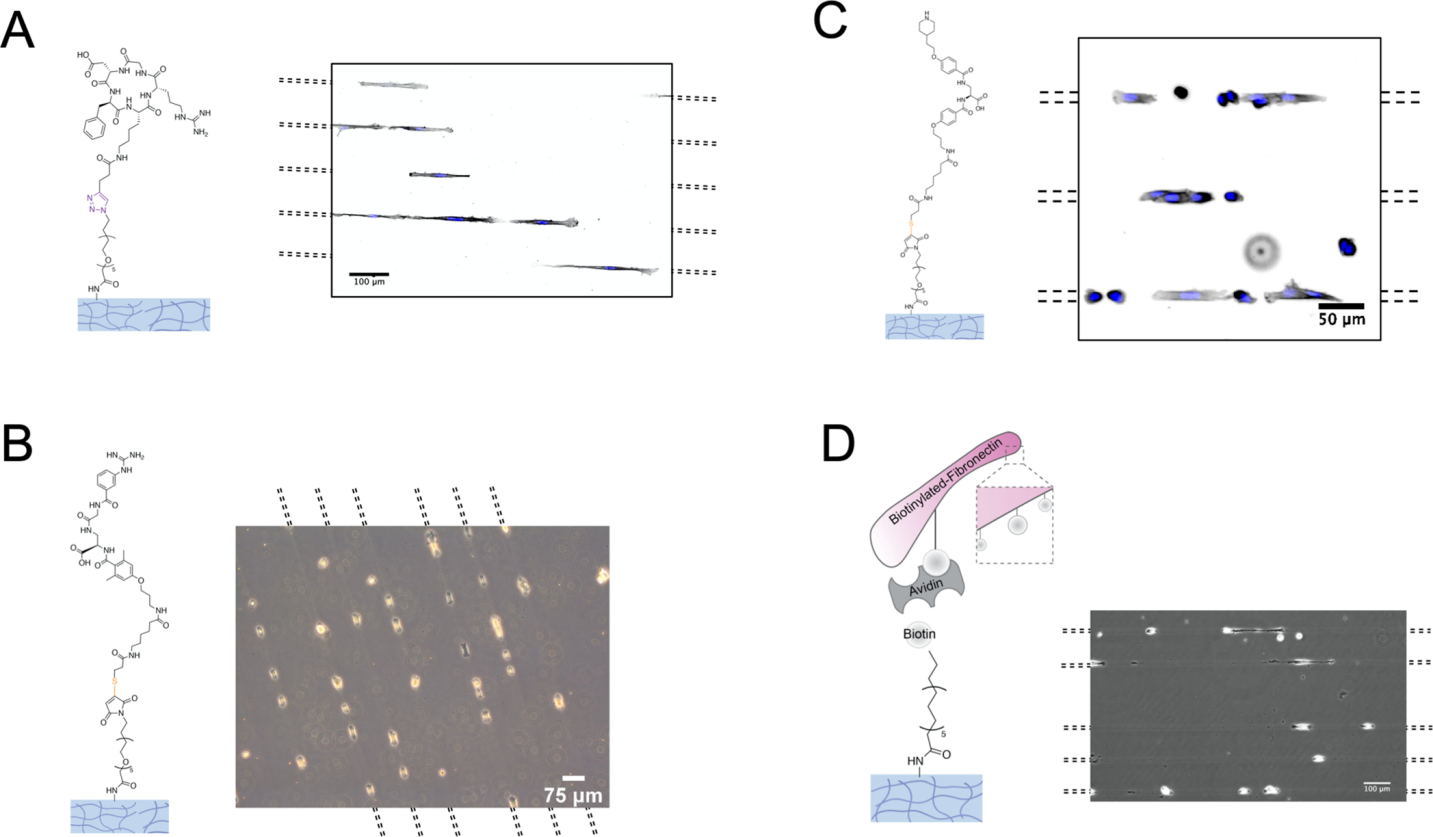
Cell patterning on poly(acrylamide) hydrogels. Line patterns were prepared on 5% Am/0.1% Bis pAAm hydrogels incorporating 3 mM caged AEMA. Patterns were functionalized with indicated integrin ligands. Primary human dermal fibroblast adhered selectively to patterns of immobilized cyclic RGDfK peptides (A) and α_5_β_1_ integrin-selective integrin peptidomimetics (B). (A) Confocal microscopy image of fixed and phalloidin-stained pHDF cells. Phalloidin stains filamentous actin; nuclei are stained in blue. (B) Phase contrast microscopy image of live pHDF cells on hydrogel patterned with α_5_β_1_ integrin-selective peptidomimetics. (C) CHO-A5 cells adhered to patterned α_IIb_β_3_ integrin-selective ligands as exemplified with the epifluorescence microscopy image of phalloidin-stained cells presented (nuclei stained in blue). (D) Phase contrast microscopy image of live pHDF recognizing line patterns of fibronectin immobilized through biotin–streptavidin chemistry. Scale bar (A,D): 100 μm, B: 75 μm, (C): 50 μm.

An unmet challenge for the majority of existing patterning methods for pAAm hydrogels is the patterned immobilization of short peptide or peptidomimetic ligands. Techniques to pattern such low-molecular-weight ligands have been reported for other hydrogel systems;^23,44,45^ here, we demonstrated as a proof of principle, the patterning in lines of integrin peptide or peptidomimetic ligands on pAAm hydrogels, and visualized cell adhesion using optical microscopy. Primary human dermal fibroblasts (pHDF) adhered selectively to patterned hydrogels functionalized using click chemistry with cyclic peptides containing the RGD peptide motif (cyclic RGDfK; Figure 4A). pHDF elongated and migrated along the lines, often exhibiting high aspect ratios of >10 (Movie S1). Small peptidomimetic integrin-selective ligands^46,47^ were also immobilized using the maleimide-thiol coupling strategy. pHDF adhered selectively to patterned areas with α_5_β_1_-selective integrin ligands (Figure 4B), while Chinese hamster ovary cells overexpressing the α_IIb_β_3_ integrin (CHO-A5) recognized α_IIb_β_3_-selective integrin ligands patterned in lines (Figure 4C). Immobilization of larger ECM proteins, such as fibronectin (FN), was also successful using the biotin-avidin linkage to confine cells in defined patterns (Figure 4D). The above results demonstrate the versatility of the presented system to pattern various ligands and the generality in application through the use of different cell types.

### Effects of Pattern Shape on Cell Behavior

**Figure 5:**
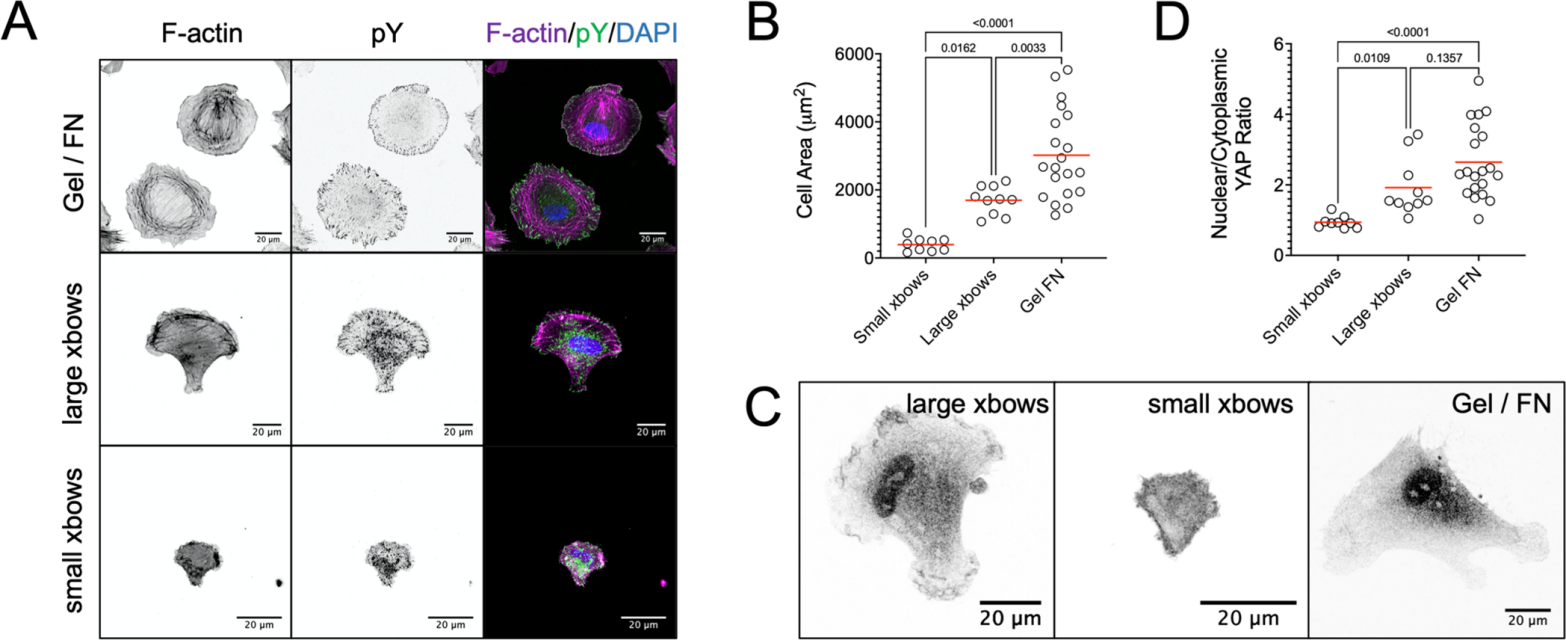
Size of cell adhesive patterns on hydrogels affects cell mechanosensing. (A) Confocal microscopy images of pHDF cells on hydrogels patterned with cyclic RGDfK peptides (small and large crossbows) or homogeneously coated FN. pHDF were fixed 4 h after seeding and stained with tetramethylrhodamine (TRITC) phalloidin, which stains filamentous actin (F-actin), and against pY, which stains focal adhesions. (B) Quantification of cell area of pHDF adhering to patterned, or FN-coated hydrogels. Each data point corresponds to a single cell, from two independent experiments. (C) Confocal microscopy images of yes-associated protein 1 (YAP) immunostained pHDF cells 4 h after seeding. (D) Quantification of nuclear-to-cytoplasmic ratio of YAP in pHDF adhering to patterned, or FN-coated hydrogels. Each data point corresponds to a single cell, from two independent experiments. Data in (B) and (D) were compared using one-way analysis of variance (ANOVA) (*n* = 9, 10, and 20 for small xbows, large xbows, and Gel FN, respectively); *P* values are shown. Scale bars 20 μm.

We next examined how the shape of micropatterned ligands affects cell mechanosensing. pHDF fibroblasts were seeded on 7 kPa hydrogels exhibiting crossbow patterns of two different sizes functionalized with cyclic RGDfK-containing peptides using click chemistry, or the same hydrogels homogeneously coated with fibronectin and the commonly used sulfo-SANPAH cross-linker. pHDF fibroblasts adhered selectively to patterns, adopting a polarized shape (Figure 5A,B). Notably, the cell size distribution was narrower on patterned substrates compared to the homogeneously coated hydrogel, demonstrating an advantage of cell patterning (Figure 5B). On the larger crossbows (50 μm in height and width), fibroblasts assembled distinct focal adhesions at their periphery as well as discernable actin stress fibers (Figure 5A). In contrast, on the smaller-sized crossbows (30 μm in height and width), fibroblasts assembled much smaller adhesions and did not exhibit an organized actin cytoskeleton (Figure 5A). Fibroblast height was also determined by pattern/cell shape; cells on the larger patterns were flatter compared to those confined to a smaller area (Figure S3).

Next, the localization of yes-associated protein 1 (YAP), a mechanosensitive transcriptional regulator that shuttles between the nucleus and cytoplasm depending on cell shape and actomyosin contractility,^48^ was examined. Previous work has demonstrated that confinement of cell size by reducing accessible adhesive area on rigid glass, or culturing of cells on soft substrates, leads to YAP nuclear export.^7^ In particular, the transition from cytosolic to nuclear YAP in isolated cells occurs in the 1–10 kPa range, with mostly nuclear YAP present on FN- or collagen-coated pAAm hydrogels with a Young’s modulus of 10 kPa.^49,50^ However, the actual threshold for nuclear accumulation/export of YAP depends not only on stiffness but also on other parameters such as ligand density.^39,51^ Here, we examined how YAP localization is influenced by the spread area on 10 kPa hydrogels. The nuclear/cytoplasmic YAP ratio was significantly higher for fibroblasts seeded on the larger patterns (Figure 5C,D). Fibroblasts adhering to homogeneously FN-coated hydrogels of the same stiffness showed mostly nuclear YAP localization as expected^49,50^ (Figure 5C,D), highlighting the effect of pattern shape on mechanotransduction. In sum, the above results demonstrate how control over the size of adhesive patterns on soft elastic hydrogels can be used to study cell behavior and open the way for studying the combinatorial effects of substrate stiffness, cell shape, and ligand type. Of particular interest is the investigation of the relative contributions of substrate stiffness and cell area on YAP mechanotransduction to test recently developed models.^52^ Moreover, while the effect of ligand density was not investigated here, the possibility to control the concentration of caged comonomer (Figure 1) and light dose (Figure 2B) can be employed to independently control the number of adhesive ligands as well.

### Traction Force Microscopy on Patterned Hydrogels

**Figure 6:**
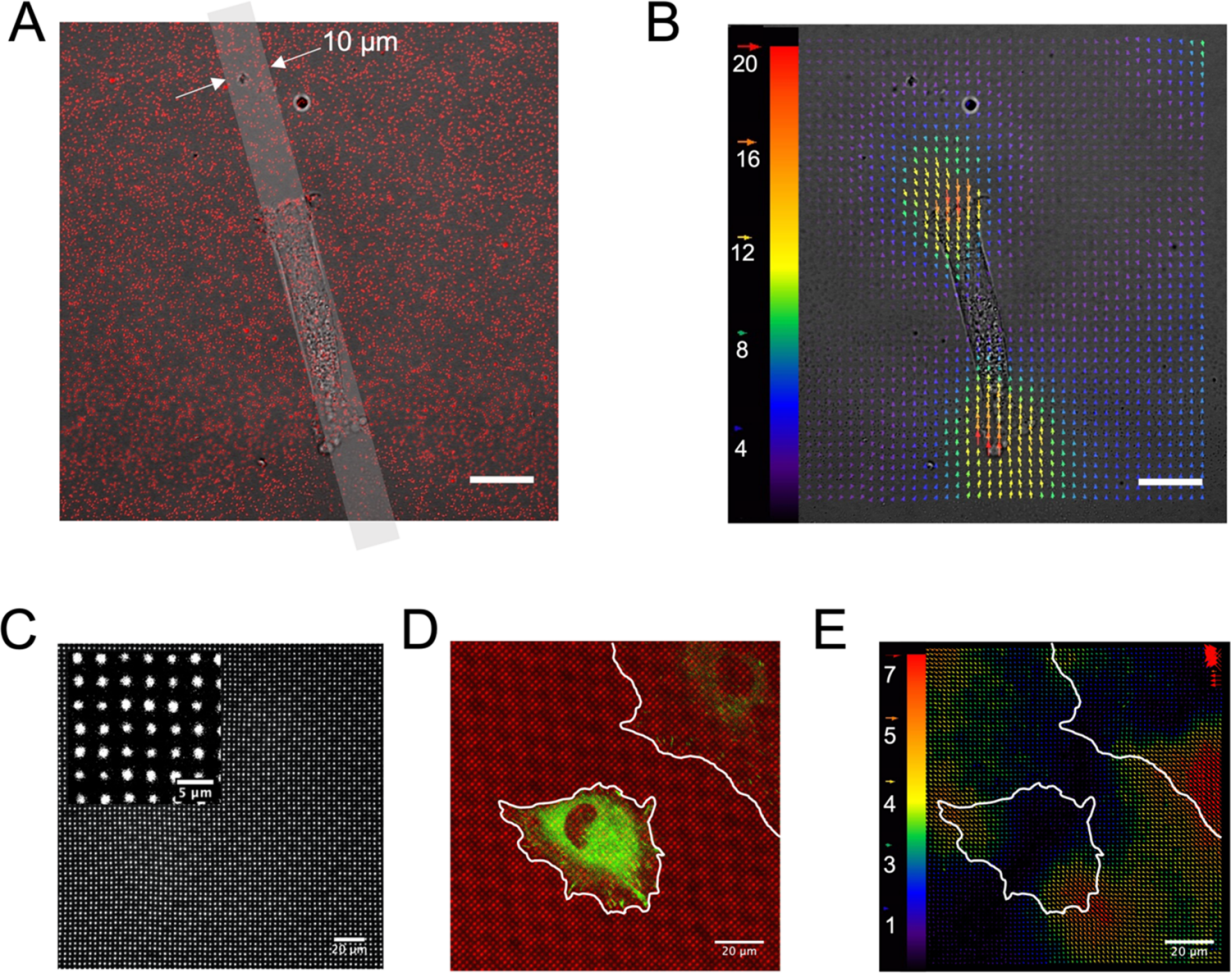
Applicability of patterned poly(acrylamide) hydrogels for traction force microscopy applications. (A) Confocal microscopy image of fluorescent beads on the upper layer of the hydrogel, merged with the transmission image showing a live pHDF fibroblast adhering on a line pattern (schematically overlaid in gray). The hydrogel used had a Young’s modulus of 2 kPa and was functionalized with α_5_β_1_ integrin-selective peptidomimetics. (B) Particle image velocimetry (PIV) analysis results from bead displacements observed before and after the removal of the cell shown in (A). The magnitude of the color-coded vectors is given in pixels. (C) Confocal microscopy image of a patterned and AlexaFluor 568-labeled hydrogel. A square grid pattern of 1 μm circles spaced 3 μm apart was used as the virtual mask. (D) Merged confocal microscopy image of a live REF_YFP-PAX_ cell with a fluorescent pattern on a 7 kPa hydrogel. (E) Substrate deformation field calculated from PIV analysis of panel (D). Cell outlines (white) in (D, E) are a guide to the eye. Scale bar: 20 μm; inset: 5 μm.

One major application of pAAm hydrogels remains traction force microscopy (TFM) for the estimation of cell-generated forces,^34^ despite the development of more physiologically relevant viscoelastic substrates or three-dimensional (3D) culture systems. In TFM, fluorescent beads embedded in the elastic substrate serve as fiducial markers that report on substrate deformations induced by cells. The confinement of cells within adhesive patterns on top of TFM substrates allows the estimation of traction forces as a function of cell shape, as well as the averaging over many cells to obtain more reliable conclusions.^4,53^ Existing protocols however rely on immobilization of large ECM proteins. To test if our patterning approach with small molecular ligands was compatible with TFM, beads were encapsulated in 2 kPa pAAm hydrogels containing caged AEMA, patterned, and functionalized with α_5_β_1_ integrin-selective peptidomimetics. The photopatterning process did not cause bleaching of the beads and pHDF fibroblasts spread and migrated along the patterned lines (Figure 6A). Importantly, standard protocols could be used to determine substrate deformations through particle image velocimetry (PIV) analysis (Figure 6B). Time lapse imaging of cells moving on patterns showed how deformations are spatially confined in the axis of the patterned lines and can be used for future calculations of exerted traction forces (Movie S2).

Traction force determination requires the reference (relaxed) state of the hydrogel, which is typically achieved following cell detachment. An alternative approach is to perform reference-free traction force microscopy using a regular pattern, which upon deformation, and hence deviation from the ideal mesh, can inform on the forces exerted on the substrate.^54^ As a proof of principle, we patterned a regular, square grid pattern of the highest resolution possible. The technical limitations of the current setup (PRIMO system with 20× objective) allowed patterning of 1 μm circular patterns, with an interspot distance of 3 μm (Figures 6C and S4). These gels were functionalized with fibronectin and sulfo-SANPAH prior to seeding a rat embryonic fibroblast cell line (REF52) that stably express paxillin-coupled to yellow fluorescent protein (REF_YFP-PAX_). Paxillin is a marker of focal adhesions in cells. An analogous analysis as for the standard TFM substrates was performed (Figure 6D,E and Movie S3), demonstrating the applicability of this approach to measure substrate deformations.

## Conclusions

The micropatterning strategy introduced here provides a versatile method to introduce cell adhesive ligands on demand, without the need to create photomasks or use harsh reaction conditions. The approach uses mild UV light to uncage reactive groups within the cross-linked polymer network; subsequent functionalization with established chemistries was demonstrated here for commonly used bioconjugation methods but can be expanded to other chemistries. Importantly, this methodology allows the patterning of low-molecular-weight ligands, a challenge with existing methods, which mostly relied on nonspecific reaction with amino acid side chains of ECM proteins. The applicability of the patterned substrates was exemplified by demonstrating the regulation of YAP activity as a function of pattern size on physiologically stiff substrates. The presented hydrogels are compatible with standard TFM protocols and could potentially be adapted for use in reference-free TFM.

## Experimental Section

### Materials

A list of commercially available reagents and antibodies used in this study are presented in the Supporting Information (Tables S1 and S2). Integrin-selective peptidomimetic ligands against integrins α_5_β_1_ and α_IIb_β_3_ containing a thiol group have been previously described.^46,47^ A short DNA sequence (5′-TTTTTTTTTTTTTTTTTTTT-3′) coupled to a thiol at the 5′ end and fluorescein to the 3′ end was purchased from biomers.net. The cyclic peptide RGDfK functionalized with an azide at the ε-amine of lysine was purchased from PSL laboratories (Heidelberg, Germany).

NVOC-protected aminoethylmethacrylamide (Caged AEMA) was prepared using a previously described protocol.^55,56^ Briefly, 2-aminoethylmethacrylamide hydrochloride (100 mg, 0.6 mmol, 1 equiv) was mixed with Na_2_CO_3_ (63.58 mg, 0.6 mmol, 1 equiv) in 16 mL of H_2_O. Then, an equimolar amount of 4,5-dimethoxy-2-nitrobenzyl chloroformate (165.38 mg, 0.6 mmol, 1 equiv) was dissolved in 16 mL of dioxane and was slowly added to the aqueous solution with vigorous stirring. After stirring at room temperature for a period of 1 h, the reaction was diluted with 15 mL of dichloromethane, followed by the addition of 10 mL of a 1 M KHSO_4_ for acidification. The organic phase was collected, and the aqueous phase was extracted with dichloromethane (3 × 15 mL). The combined organic extracts were dried over anhydrous MgSO_4_, and concentrated *in vacuo* to give 142 mg (66%) of a yellowish solid, which was used without further purification. The product was conserved, at −20 °C, protected from light.

^1^H NMR (400 MHz, CDCl_3_) δ 7.68 (s, 1H), 6.98 (s, 1H), 6.49 (br, 1H), 5.69 (s, 1H), 5.48 (s, 2H), 5.30 (s, 1H), 3.95 (s, 3H), 3.94 (s, 3H), 3.69 (s, 1H), 3.46 (t, *J* = 6 Hz, 2H), 3.40 (t, *J* = 6 Hz, 2H), 1.91 (s, 3H). ^13^C NMR (101 MHz, CDCl_3_) δ 169.18, 156.90, 153.58, 148.13, 139.75, 139.41, 127.99, 120.18, 110.07, 108.14, 67.09, 63.73, 56.43, 41.14, 40.66, 18.55. HRMS (ESI) *m*/*z* [M + H]^+^, calculated = 368.1452 (calculated for C_16_H_21_N_3_O_7_), observed = 368.1447, 1.36 ppm.

### Hydrogel Preparation

Poly(acrylamide) hydrogels were prepared using radical polymerization according to a published protocol.^35^ The weight percent of acrylamide monomer was kept constant at 5%, while the weight percent of the cross-linker bis-acrylamide was varied between 0.03 and 0.3%. Comonomers were introduced in the precursor solution at defined concentrations. 2-Aminoethylmethacrylamide (AEMA) was introduced as an aqueous solution, while caged AEMA was introduced in DMF. Fluorescent beads, 200 nm in diameter, were added (1:100 stock solution) to the precursor mixture prior to gelation. After mixing, 1 μL of tetramethylethylenediamine (TEMED) and 15 μL of freshly prepared solution of ammonium persulfate (APS; 100 mg/mL) were added and the precursor solution was vortexed and pipetted between a hydrophobic glass coverslip, treated with Rain-X for 10 min, and a glass coverslip treated with (3-aminopropyl)triethoxysilane (APTES). For silanization, the coverslips were immersed for 1 h in 100 mL of an APTES solution in ethanol (10% v/v), in which 10 μL of water and 10 μL of triethylamine were added. Then, the coverslips were rinsed with ethanol, then water, dried, and placed in an oven at 120 °C for 1 h. The hydrogel thickness was controlled using spacers between the two coverslips. Gelation was left to proceed for at least 30 min at room temperature. Gels attached to the APTES-treated coverslips were recovered after removing the hydrophobic coverslip and were washed in excess of water five times to remove unreacted monomers and initiators.

### Mechanical Characterization

Hydrogels were mechanically characterized by indentation measurements using a Nano-Wizard III atomic force microscope (AFM; JPK Instruments AG, Germany), mounted on an optical microscope (Zeiss Axiovert200). Cantilevers with a spherical, borosilicate glass probe 5 μm in diameter (sQube) and a spring constant between 0.45 and 0.60 N/m were used. The exact value of the spring constant was determined using the thermal noise calibration method prior to measurements. Force–distance (F–d) curves were obtained from immobilized gels with a cantilever speed of 1.0 μm/s in phosphate-buffered saline (PBS) at room temperature. Young’s moduli were calculated by fitting the F–d curves using the software provided by JPK and the Hertz model for a spherical indenter: , where *E* stands for Young’s modulus, ν for the Poisson’s ratio of the sample, *R* is the radius of the indenting sphere, δ is the indentation depth, and δ_0_ is its zero position. A Poisson ratio of 0.5 was used. At least 30 curves from at least three different positions were analyzed per hydrogel.

### Comonomer Incorporation

The amount of incorporated AEMA was determined using the amine-reactive dye fluorescamine. Hydrogels with different AEMA concentrations were formed in 96-well plates (50 μL/gel) for 30 min and then extensively washed with water. Water (100 μL) was added on top of each gel, and 5 μL of a fluorescamine solution in dimethyl sulfoxide (DMSO) (3.0 mg/mL) was added. The well plate was shaken for 10 min, and fluorescence intensity was measured using a TECAN well plate reader (excitation wavelength: 390 nm; emission wavelength: 470 nm). AEMA concentrations were calculated using a calibration curve obtained from aqueous comonomer solutions.

The amount of incorporated caged AEMA in hydrogels formed in 96-well plates was determined by UV absorption measurements at a wavelength of 350 nm, using a TECAN well plate reader. Precursor solutions without the comonomer were used as blanks.

### Micropatterning

Hydrogels were patterned using the PRIMO system (Alveole) on an inverted Nikon Eclipse Ti2-E equipped with a 20×/0.5 NA objective. Patterns (virtual masks) were designed using the software Inkscape and were saved as 8-bit Tiff files (256 levels of gray value). The designed patterns were then loaded on the Leonardo software, the objective was focused on the hydrogel surface, and the UV dose was selected. After illumination, hydrogels were washed five times for 5 min with water prior to functionalization and were protected from light.

### Hydrogel Functionalization

Micropatterned hydrogels were equilibrated in 10 mM PBS prior to the reaction of deprotected primary amines with heterobifunctional linkers. Four strategies to functionalize the hydrogels were performed.

(1) Hydrogels were first reacted with 1 mM sulfosuccinimidobiotin (NHS-biotin) for 30 min at room temperature, washed three times for 5 min with water and two times for 5 min with PBS, and incubated with 100 μg/mL streptavidin or Atto565-labeled streptavidin for 1 h at room temperature. Unbound streptavidin molecules were removed by washing four times for 5 min with PBS and hydrogels were incubated with 40 μg/mL biotinylated FN overnight at 4 °C. Hydrogels were washed three times for 5 min with PBS prior to cell seeding.
(2) Hydrogels were first reacted with 4.7 mM succinimidyl-[(*N*-maleimidopropionamido)-diethyleneglycol] ester (NHS-PEG_2_-maleimide) for 10 min at room temperature, washed five times for 10 s with PBS, and incubated with thiol-containing integrin peptidomimetics (100 μM) or thiolated DNA (10 μM) for 1 h at room temperature. Hydrogels were washed five times for 5 min with water prior to imaging, or three times for 5 min with water and two times for 5 min with PBS prior to cell seeding.
(3) Hydrogels were first reacted with 2 mM Azide-PEG_4_-*N*-hydroxysuccinimidyl ester (Azide-PEG_4_-NHS) for 30 min at room temperature and washed three times for 5 min with water and two times for 5 min with PBS. Then, a click reaction with an alkyne-coupled AlexaFluor 555 dye (0.5 mM) was performed in 4-(2-hydroxyethyl)-1-piperazineethanesulfonic acid (HEPES) buffer (100 mM) containing 2 mM Cu_2_SO_4_ and 5 mM sodium ascorbate. Hydrogels were washed five times for 5 min with water prior to imaging.
(4) Hydrogels were first reacted with 2.5 mM Alkyne-PEG_5_-*N*-hydroxysuccinimidyl ester for 30 min at room temperature and washed three times for 5 min with water and two times for 5 min with PBS. Then, a click reaction with 100 μM cyclic RGDfK peptide containing an azide group, or 100 μM azide-coupled AlexaFluor488, was performed in HEPES buffer (100 mM) containing 2 mM Cu_2_SO_4_ and 5 mM sodium ascorbate. Hydrogels were washed five times for 5 min with water prior to imaging, or three times for 5 min with water and two times for 5 min with PBS prior to cell seeding.

Hydrogels were alternatively functionalized using the standard approach with sulfosuccinimidyl-6-[4′-azido-2′-nitrophenylamino]hexanoate (suflo(SANPAH)). Briefly, hydrogels were washed with PBS before applying a 1 mg/mL sulfo(SANPAH) solution in HEPES buffer (100 mM, pH 7.4). The hydrogel was then irradiated with a portable UV light lamp for 5 min and rinsed first with water and then with PBS. Finally, a fibronectin solution in PBS (40 μg/mL) was placed on top of the hydrogel and incubated overnight at 4 °C. Hydrogels were washed three times with PBS prior to cell seeding.

### Cell Culture

Primary human dermal fibroblasts (pHDF) were purchased from ATCC (Cat #PCS-201-010). Chinese hamster ovary cells overexpressing the α_IIb_β_3_ integrin (CHO-A5) were a kind gift from the lab of Prof. M. Ginsberg (UCSD).^57^ REF52 stably transfected with paxillin fused to a yellow fluorescent protein (REF_YFP-PAX_) was a gift from Prof. B. Geiger (Weizmann Institute). All cells were cultured as sub-confluent monolayers at 37 °C and 5% CO_2_, in Dulbecco’s modified Eagle’s medium (DMEM; Life Technologies; Prod. #10938), supplemented with 10% fetal bovine serum (FBS) and 1% penicillin/streptomycin (P/S). pHDF cultures were used until passage 15. Cell cultures were checked regularly for the absence of mycoplasma.

### Immunofluorescence and Microscopy Imaging

Cells were fixed on hydrogels after washing once with PBS and incubating with a 4% paraformaldehyde (PFA) PBS solution for 20 min at room temperature. After washing with PBS, the cells were permeabilized with 0.1% Triton X-100 for 5 min, followed by blocking with 1% bovine serum albumin (BSA) in PBS for 1 h. The cells were incubated with primary antibodies (diluted in 1% BSA) for 1 h at room temperature, washed four times with PBS, and incubated with corresponding secondary antibodies for another 1 h at room temperature. 4′,6-Diamidino-2-phenylindole (DAPI) and TRITC-phalloidin were used to stain nuclei and filamentous actin (F-actin), respectively.

Laser scanning confocal microscopy was performed on a Zeiss LSM880 microscope equipped with a 20×/0.8 NA air objective and a 40×/1.1 NA water immersion objective. Epifluorescence and phase contrast microscopy experiments were performed on a Leica DMi8. Live cell microscopy was performed at 37 °C and 5% CO_2_ using environmental chambers.

### Hydrogel Deformations

Confocal microscopy images of patterned hydrogels with embedded beads or with fluorescent square grid patterns (AlexaFluor 568-labeled) were acquired in the presence of cells and after cell death induced by the addition of 100 μL of 1 N NaOH solution in 5–6 mL of cell culture medium. Cell death/removal was verified by visual inspection. The two images (before–after) were aligned using the StackReg plugin of ImageJ (http://bigwww.epfl.ch/thevenaz/stackreg/). Then, particle image velocimetry (PIV) analysis was performed using the PIV plugin (https://sites.google.com/site/qingzongtseng/piv).

## Supporting Information Available

List of reagents (Table S1) and antibodies (Table S2); scheme of poly(acrylamide) hydrogel synthesis (Figure S1); confocal microscopy z-stack of labeled patterns (Figure S2) and stained cells (Figure S3); fluorescence images of square patterns on hydrogels (Figure S4) (PDF)
Time lapse phase contrast imaging of pHDF migrating along micropatterned lines (Movie S1) (AVI)
Time lapse imaging of cells on a standard TFM substrate moving along a line pattern (Movie S2) (AVI)
Time lapse imaging of REF_YFP-PAX_ on FN-coated hydrogels that were micropatterned with a square fluorescent grid (Movie S3) (AVI)

## Supplementary Material

am1c17901_si_001.pdf

am1c17901_si_002.avi

am1c17901_si_003.avi

am1c17901_si_004.avi
