# Supplementary material for: Facile and Versatile Method for Micropatterning Poly(acrylamide) Hydrogels Using Photocleavable Comonomers
Source: ACS Appl Mater Interfaces. 2022 Jan 10;14(3):3643–52. doi: 10.1021/acsami.1c17901 (PMC8796170; doi:10.1021/acsami.1c17901)
Supplement: Supplementary file 1 — am1c17901_si_001.pdf [file am1c17901_si_001.pdf]

Supporting Information

A facile and versatile method for micropatterning poly(acrylamide) hydrogels using photocleavable co-monomers.

Dimitris Missirlis[1], Miguel Baños[1], Felix Lussier[1] and Joachim P. Spatz[1,2]

[1] Max-Planck-Institute for Medical Research, Department of Cellular Biophysics, Jahnstr. 29, Heidelberg 69120, Germany

[2] Heidelberg University, Department of Biophysical Chemistry, Physical Chemistry Institute, INF-253, Heidelberg 69120, Germany

[*] corresponding author: email: dimitris.missirlis@mr.mpg.de, tel: +49 6221 486430

Table S1. Reagents used in our study

Reagent	Abbreviation	Supplier	Cat. No.
**Acrylamide solution**	Am	Sigma	A4058
**N,N' methylenebis(acrylamide)**	Bis	Sigma	M1533
**Bovine Serum Albumin**	BSA	Sigma-Aldrich	A4161
**2-aminoethyl methacrylate**	-	Sigma	516155
**Ammonium Persulfate**	APS	Sigma	A3678
**2-aminoethyl methacrylamide**	AEMA	Sigma	900652
**Sodium-L-Ascorbate**	TEMED	Sigma	A4034
**N,N,N′,N′-Tetramethyl ethylenediamine**	-	Sigma	T9281
**Bovine Plasma Fibronectin**	FN	Thermo Scientific	33010018
**Biotin-labeled Fibronectin**	biotin-FN	tebu-bio	FNR03
**4% Paraformaldehyde in PBS**	PFA	Santa Cruz	sc-281962
**Phalloidin-tetramethylrhodamine B isothiocyanate**	TRITC-Phalloidin	Sigma-Aldrich	P1951
**4',6-Diamidino-2-Phenylindole, Dihydrochloride**	DAPI	Thermo-Fisher	D1306
**Fluospheres™ Carboxylate-Modified Microspheres, 0.2 µm, red fluorescent**	-	Thermo-Fisher	F8810
**Triethylamine**	-	Sigma-Aldrich	471283
**(3-Aminopropyl)triethoxysilane**	APTES	Sigma-Aldrich	A3648
**Triton X-100**	-	Sigma-Aldrich	T7878
***N*-(3-Dimethylaminopropyl)-*N′*-ethylcarbodiimide hydrochloride**	EDC	Sigma-Aldrich	03450
**Dimethyl sulfoxide**	DMSO	Sigma-Aldrich	D8418
**Dimethyl formamide**	DMF	Merck	103053
**Ethanol**	-	Roth	9065.5

**Sulfosuccinimidobiotin**	NHS-biotin	Thermo Scientific	21326
**succinimidyl-[(*N*-maleimidopropionamido)-diethyleneglycol] ester**	NHS-PEG$_2$-Maleimide	Thermo Scientific	22102
**Azide-PEG4-*N*-hydroxysuccinimidyl ester**	NHS-PEG$_4$-Azide	Thermo Scientific	26130
**Alkyne-PEG5-*N*-hydroxysuccinimidyl ester**	Alkyne-PEG$_5$-NHS	Sigma	764191
**Streptavidin**	-	Sigma	S4762
**Atto 565 Streptavidin**	-	Sigma	56304
**AlexaFluor 555 Alkyne**	-	Thermo Scientific	A20013
**AlexaFluor 488 Azide**	-	Jena Biosciences	CLK-1275-5
**Cu_2SO_4**	-	Grüssing	12079
**Fluorescamine**	-	Sigma	F9015
**AlexaFluor 568 NHS ester**	-	Thermo Scientific	A20003
**Atto 647N NHS ester**	-	Sigma	18373
**Sulfosuccinimidyl-6-[4'-azido-2'-nitrophenylamino]hexanoate**	Sulfo(SANPAH)	Thermo Scientific	A35395

Table S2. Antibodies used in our study

Antibody	Clone	Application / Dilution	Supplier	Cat. No.
anti-YAP	D8H1X	Immunofluorescence / 1:100	Cell Signaling	14074
anti-pY	PY99	Immunofluorescence / 1:100	Santa Cruz Biotechnology	sc-7020
anti-mouse IgG AlexaFluor488 conjugate	polyclonal	Immunofluorescence / 1:150	Thermo-Fisher	A11001
anti-rabbit IgG AlexaFluor647 conjugate	polyclonal	Immunofluorescence / 1:150	Thermo-Fisher	A21244

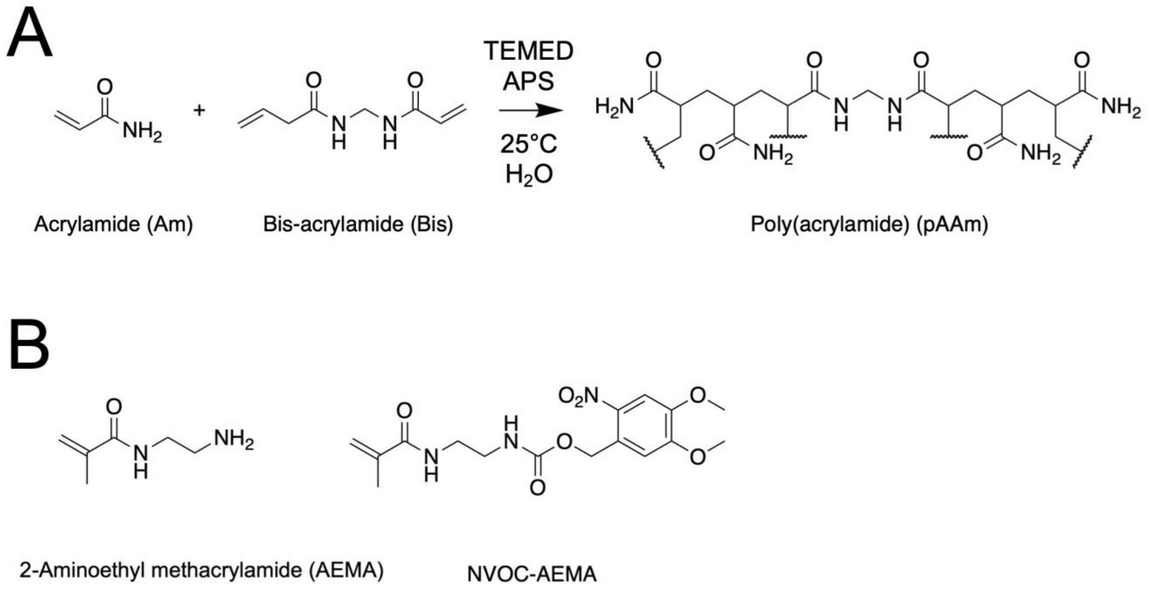

Figure S1. Schematic of the radical cross-linking polymerization reaction for poly(acrylamide) hydrogel formation. A) Acrylamide (Am) and Bis-acrylamide (Bis) are mixed in different ratios and addition of ammonium persulfate (APS) and TEMED initiate the vinyl-addition, radical polymerization. **B)** Chemical structure of 2-aminoethyl methacrylamide (AEMA) and the newly synthesized AEMA capped with an NVOC group (see experimental section for details of the reaction).

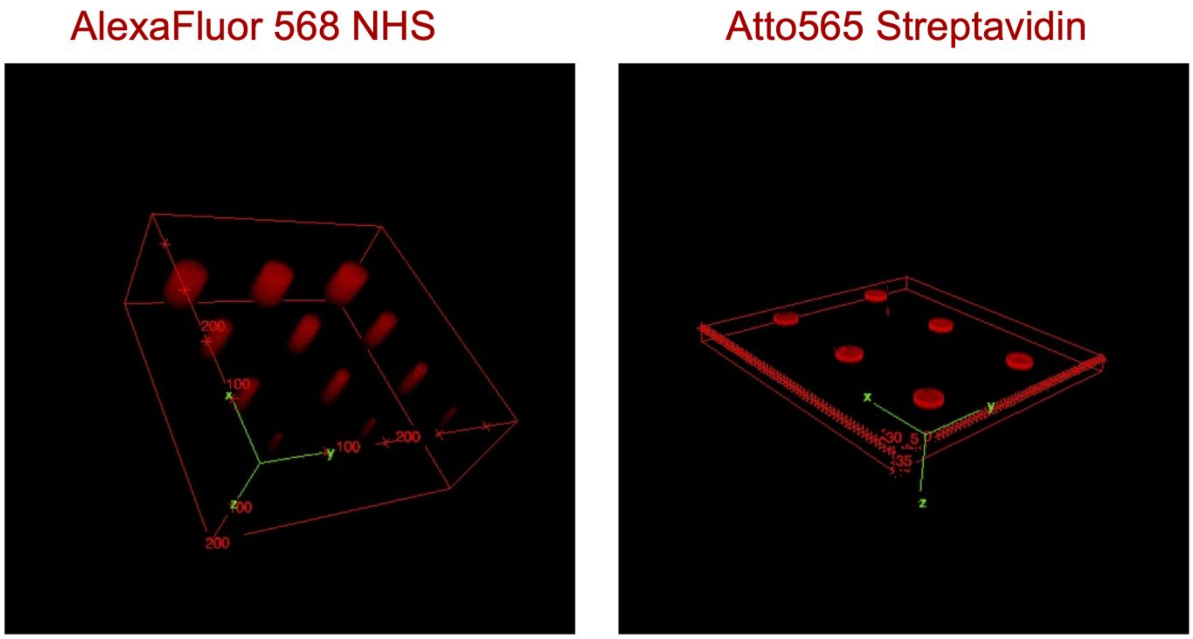

Figure S2. Confocal microscopy z-stacks of patterned hydrogels. Poly(acrylamide hydrogels) containing caged AEMA (1 mM) were patterned and reacted with NHS-AlexaFluor568 (Left) or NHS-biotin and Atto565-Streptavidin (Right). Z-stacks revealed incorporation of the dye throughout the hydrogel thickness for the small AlexaFluor dye, whereas fluorescence was only observed at the hydrogel surface for the larger, labeled streptavidin molecules. In both cases, the dye was confined to the patterned areas.

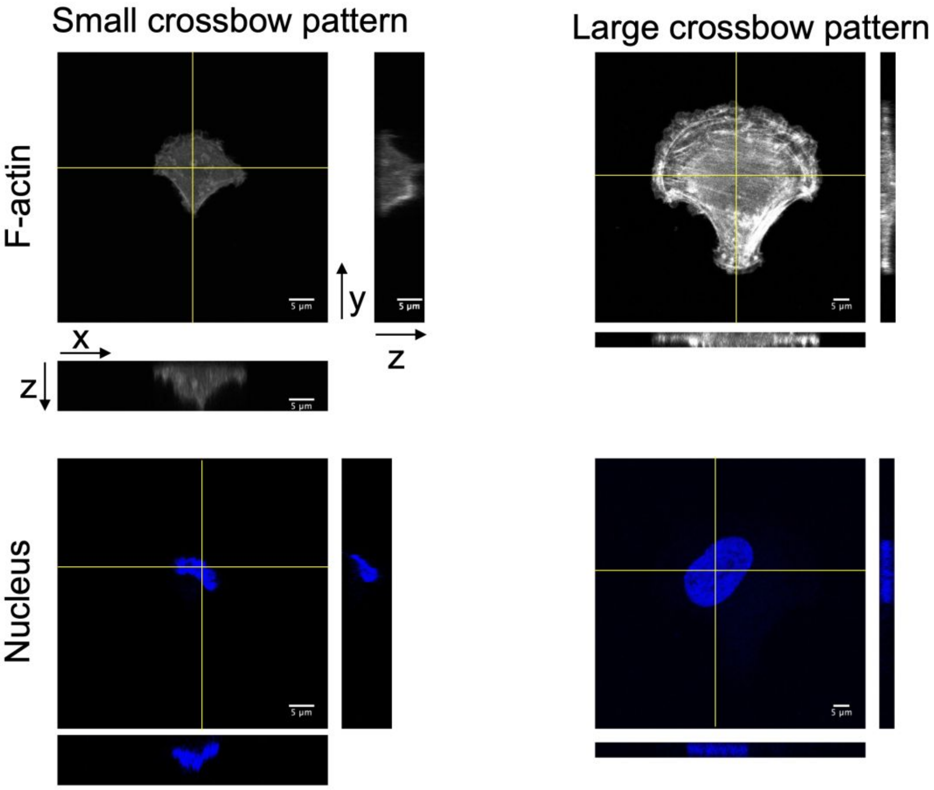

Figure S3. pHDF fibroblast height is controlled by the size of adhesive pattern on hydrogels. Orthogonal views reconstructed from z-stack confocal imaging of fixed pHDF cells stained with phalloidin and DAPI. Cells were seeded on 7 kPa hydrogels patterned with small (30x30 µm) or large (50x50 µm) crossbow patterns and fixed 4 hours post-seeding.

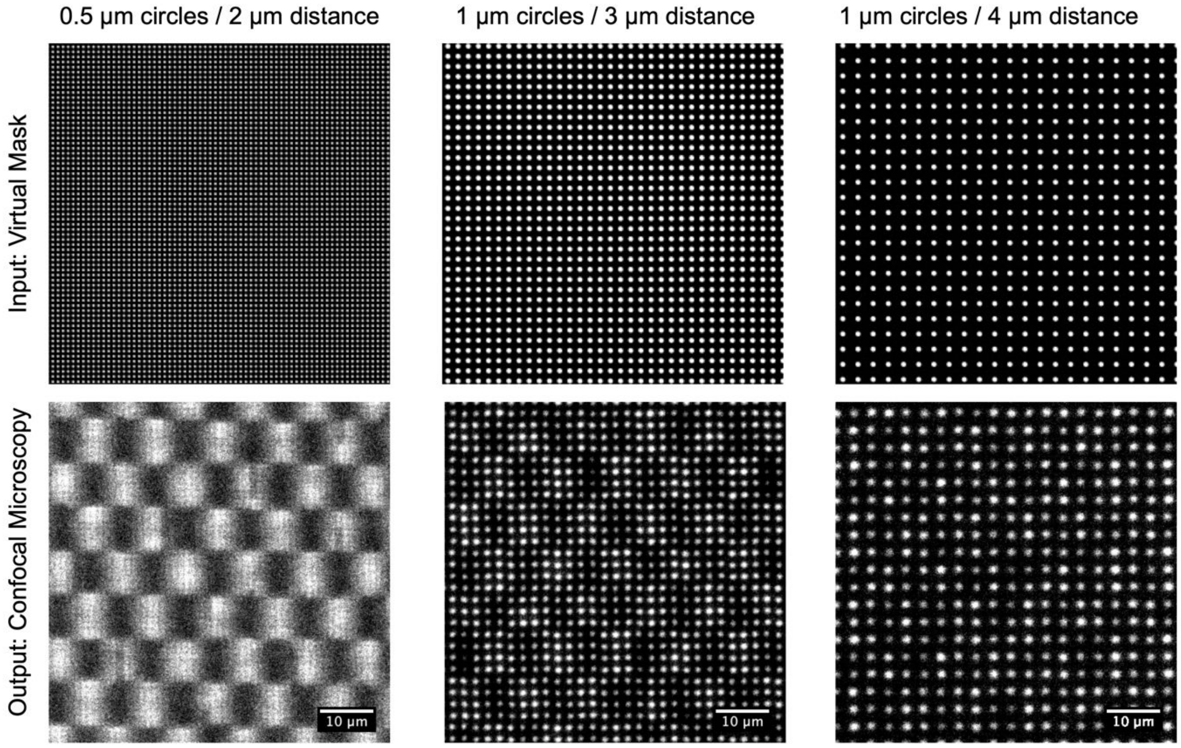

Figure S4. Square grid pattern designs. Different virtual masks were designed with circular shapes of 0.5 or 1.0 µm in diameter and spaced 2, 3 or 4 µm apart (center-center distance) and used for micropatterning 3 kPa pAAm hydrogels containing 1 mM caged AEMA. After patterning hydrogels were reacted with NHS-AlexaFluor 568 and visualized using confocal microscopy. The smallest features (0.5 µm circles) were not successfully resolved.
